# POMSNAME: an aide-mémoire to improve the assessment and documentation of palliative care – a longitudinal project

**DOI:** 10.1186/s12904-023-01279-1

**Published:** 2023-10-21

**Authors:** Ann Dadich, Martyna Gliniecka, Michelle Cull, Kerrie Womsley

**Affiliations:** 1 School of Business, Western Sydney University, Locked Bag 1797, Penrith South, NSW 2751 Australia; 2 Illawarra Shoalhaven Local Health District, PO Box 239, Port Kembla, NSW 2505 Australia

**Keywords:** Palliative care, Needs assessment, Documentation, Nurses, community health, Quality improvement, Delivery of health care

## Abstract

**Background:**

Evidence-based palliative care requires comprehensive assessment and documentation. However, palliative care is not always systemically documented – this can have implications for team communication and patient wellbeing. The aim of this project was to determine the effectiveness of an aide-mémoire – POMSNAME – to prompt the comprehensive assessment of the following domains by clinicians: pain, orientation and oral health, mobility, social situation, nausea and vomiting, appetite, medication, and elimination.

**Methods:**

A placard depicting the aide-mémoire was distributed to community-based nurses who received training and support. The case notes of palliative care patients were evaluated one month before the intervention, and was repeated at one month, eight months, and fifty months following the intervention. The 235 case notes pertained to patients who received palliative care from a team of 13 registered nurses at one community health service.

**Results:**

The documented assessment of palliative care patients improved across all nine domains. The most significant improvements pertained to patients’ social situation, orientation, and nausea, eight months after the aide-mémoire was introduced (170.1%, 116.9%, and 105.6%, respectively, all at *p* < .001). Although oral health and medication assessment declined one-month after the aide-mémoire was introduced (-41.7% and-2.1%, respectively), both subsequently improved, thereafter, at both 8 months and 50 months after the aide-mémoire was introduced.

**Conclusions:**

The improvement of palliative care documentation across all nine domains demonstrates the potential of the POMSNAME aide-mémoire to prompt the comprehensive assessment of patients by clinicians with generalist expertise. Research is required to determine whether other domains warrant inclusion and how.

**Supplementary Information:**

The online version contains supplementary material available at 10.1186/s12904-023-01279-1.

## Background

Delivered beyond a hospital, community-based palliative care can cater to the needs and preferences of patients who require palliative care and their carers [1]. Delivered across different contexts, including offices, clinics, long-term care services, and patient homes [2], community-based palliative care can offer holistic and timely care to patients and carers, enabling patients to manage symptoms and die at home, with support [3–6]. This in turn can increase quality of life, ease the burden of care, and reduce the growing costs of healthcare [7].

The assessment and documentation of patient needs and preferences represent an important part of palliative care [8, 9]. Assessment and documentation can serve to: ascertain patterns and changes in patient needs and preferences; identify opportunities to improve the quality of life; improve communication between different individuals involved in the patient’s care; reduce duplicative or redundant efforts; and contribute to clinical credibility [10]. Many validated palliative care assessment tools are designed for self-assessments, including the Edmonton symptom assessment system [11], Palliative outcome scale – Parkinson’s disease [12], the quality care questionnaire – palliative care [13], and the PER2SON score [14].

Despite their importance, the assessment and documentation of patient needs and preferences can be challenging. This is largely due to two key reasons. First, many palliative care assessment tools are complex and lengthy, which can be taxing for patients and clinicians, alike [15]. Consider, for instance, the resident assessment instrument-home care (RAI-HC) – an assessment tool to measure patients’ health status, need for care, and information on housing and informal carers [16], comprised of some 22 to 29 items. Second, given the validity and reliability of some assessment tools – like the palliative care outcomes collaboration symptom assessment scale (PCOC SAS, 17) – they can fail to accommodate the often opportunistic and conversational ways of building rapport with a patient and carer, which are pivotal to palliative care [17]. The PCOC SAS is designed for both self-assessment and clinician assessment, focussing on symptom assessment, distress levels, and patient daily functional performance. Given its quantitative approach, comprised of different scales and scores per category, it can detract from a conversational approach with a patient or carer. This is noteworthy because death can be a taboo topic for some [18, 19]. Furthermore, palliative care is not the sole domain of palliative care specialists, but is delivered by primary care and community health clinicians with generalist expertise [20, 21] who have called for further education in symptom and pain management [22]. It is therefore important to provide clinicians with an array of evidence-based resources that are user-friendly and flexible to accommodate different forms of expertise and different approaches to patient care. Informed by the revised standards for quality improvement reporting excellence [23], this article reports on a longitudinal project to improve the assessment and documentation of palliative care.

## Methods

**Fig. 1:**
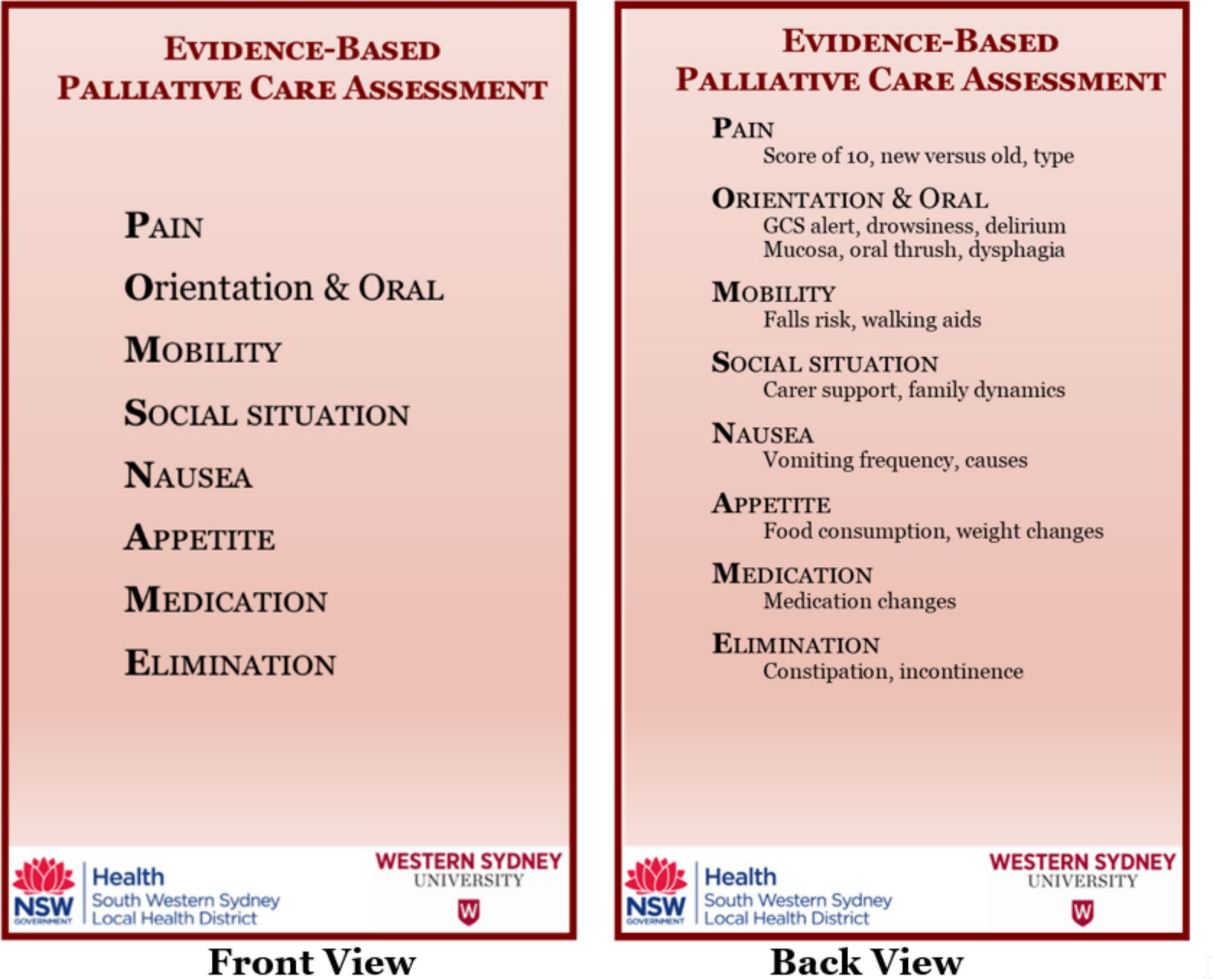
POMSNAME Aide-Mémoire

To guide – rather than dictate – palliative care assessment and documentation, an aide-mémoire was devised for community health clinicians who support patients requiring palliative care. This aide-mémoire was expected to improve the assessment and documentation of palliative care and was evaluated, accordingly. An aide-mémoire was purposely used because of its demonstrated value, particularly when assessing symptoms [24, 25]. Extant literature suggests that particular domains warrant consideration when attending to patients’ palliative care needs. These include physical and psychosocial domains [8]. Although interrelated, the former include ‘appetite… nausea… pain’ [26], falls [27], oral health [28, 29], (im)mobility [30, 31], medication regime [32], and constipation [33], while the latter refer to ‘Involving and Supporting the Family’ [3] and ‘informal carers’ [34]. Informed by this literature, the aide-mémoire serves to remind clinicians of the need to enquire about a patient’s: pain, orientation and oral health, mobility, their social situation, nausea, appetite, medication, and elimination (see Fig. 1). To optimise accessibility, the aide-mémoire was produced on a double-sided placard that can be worn on a lanyard or keychain.

The aide-mémoire was introduced to all the 13 community health nurses in a public community health centre who were responsible for providing palliative care. Located in a culturally and linguistically diverse part of New South Wales, Australia [35], the centre was part of a large local health district with a population of approximately 966,450 people [36]. The centre employed registered nurses on a rotating roster, seven days per week, who were responsible for providing palliative care, prevention, early intervention and community-based treatment, as well as rehabilitation services. Although the number of patients supported by the centre varied, approximately 300 typically received community healthcare from this centre, with approximately one-third receiving palliative care.

Key improvement areas and corresponding steps in the improvement intervention are summarised (see Table 1). The 13 community health nurses each received a placard that presented the aide-mémoire, accompanied by formal and informal training. To align with routine organisational practices within the community health centre (and thereby minimise disruption), the former included an in-service workshop (of approximately an hour) to: describe and justify the nine domains; and demonstrate how the aide-mémoire can be introduced and used during patient consultations, which were typically home visits. The latter included: regular mentoring between senior nurses (a clinical nurse consultant and three clinical nurse specialists in palliative care) and generalist clinicians; experienced generalist nurses who role modelled how the aide-mémoire can be used to less experienced generalist nurses; as well as the weekly case review meeting, during which the senior nurses opportunistically reminded generalist clinicians of the importance of the domains. It is important to reinforce that training was multimodal and ongoing, rather than a discrete project phase. This approach served to embed the aide-mémoire into routine palliative care practices, enabling it to become business-as-usual among nurses who developed greater ownership of it. This was demonstrated by the ways in which generalist clinicians introduced the aide-mémoire to clinicians who recently joined the team, including new graduate nurses.

**Table 1:** Key improvement areas and specific interventions

Key Improvement Area	Improvement Step	Improvement Group
• Patient assessment during consultation • Documentation of patient assessment in case notes	1. POMSNAME aide-mémoire presented on placards for lanyard or keychain 2. In-service workshop to describe and justify domains to be addressed during patient consultations 3. Regular mentoring by senior clinicians 4. Role modelling by experienced nurses 5. Reminder at weekly case review meetings	Community health nurses who delivered palliative care

Quality improvement methods and statistical processing were used to assess the aide-mémoire as an improvement intervention on community-based palliative care assessment and documentation. The measurement period was from September 1, 2015, to November 30, 2019 (inclusive). An evaluation [37] was conducted of patient case notes, as informed by related research, to determine the frequency of reference to each of the nine POMSNAME domains. This involved reviewing all the palliative care case notes that the 13 community health nurses documented during the project period. The nurses were registered with the national regulation agency, had received the aforesaid training, and were employed at the centre for the duration of the project. The case notes pertained to patients who were referred to the community health centre for palliative care and received such care during the project period – they were identified via the electronic medical records. The case notes were reviewed to determine the presence (or absence) of documentation pertaining to the nine domains and indicating these instances within an Excel file – when a domain had been documented, ‘1’ was indicated within the Excel file and when a domain had not been documented, ‘0’ was indicated. There were no missing data.

The evaluation of case notes occurred one month before (t=-1) the intervention (*n* = 56), as a baseline measure, and was repeated at one month (t = 1, *n* = 49), eight months (t = 8, *n* = 85), and fifty months (t = 50, *n* = 45) following the intervention – these periods served to determine the short-, medium-, and long-term effects. The frequency of occurrence for each domain in the case notes was tallied and weighted to the number of baseline cases (*n* = 56) for each time interval for comparative value. Each time interval (t = 1, t = 8, and t = 50) was then compared with the baseline using *t*-tests.

Given the importance of assessment and documentation for all patients requiring palliative care [38], irrespective of their demographic attributes or health issues, these were not considered; as such, the project involved evaluating the case notes of different patients and different timepoints. Furthermore, the audit did not involve tracking the same clinicians or patients over time – this was because: different community health clinicians were involved in a patient’s care; and some patients were discharged from the palliative care service because they no longer required community healthcare, typically due to death or a hospital admission – this accounts for the disparate numbers of case notes that were evaluated across the timepoints during the project period, ranging from 45 at fifty months, to 85 at eight months. However, the different case notes documented by different clinicians were deemed to be comparable given the uniformity of training provided to all. To optimise robustness, two auditors reviewed the patient case notes and discrepancies were reconciled via discussion with a third auditor. As a quality improvement project that met the definition of quality assurance and evaluation, as per the National Health and Medical Research Council [37], the approval of a human research ethics committee and informed consent were deemed unnecessary according to this national protocol. This was because: the case notes that were evaluated were ‘coincidental to standard operating procedures with standard equipment and/or protocols’; this project was ‘expressly for the purpose of maintaining standards or identifying areas for improvement’; the case notes were ‘not linked to individuals’; and ‘None of the triggers for consideration of ethical review’ were present. Given the project involved the examination of patient case notes, it was performed in accordance with the Declaration of Helsinki. The project was considered by the Primary and Community Health Quality and Safety Committee within the local health district, which recognised this as a quality assurance project.

## Results

Overall, palliative care assessment and documentation improved following use of the aide-mémoire, with patient notes demonstrating greater reference to most domains (see Table 2). Overall, the documentation of the nine domains improved by 28.5% at one month (t = 1); 60.8% at eight months (t = 8); and 47.5% at fifty months (t = 50).

The *t*-tests showed a statistically significant improvement at the 0.05 level in the mean reference to the domains at all post-intervention timepoints. Furthermore, at eight months (t = 8), each of the nine individual domains were documented at a significantly higher rate than the baseline, with eight domains at *p* < .001, and one (elimination) at *p* < .01. A statistically significant improvement was also found at one month (t = 1) for three domains, and at fifty months (t = 50) for seven of the domains.

**Table 2:** Comparison of POMSNAME implementation at different timepoints

Weighted Cases (*n = 56*)	Baseline	1 month post	Difference to baseline	% Improvement	8 months post	Difference to baseline	% Improvement (t = + 8)	50 months post	Difference to baseline	% Improvement (t = + 50)
	(t=-1)	(t = + 1)		(t = + 1)	(t = + 8)			(t = + 50)		
Pain	42.00	47.83	5.83	13.89%	56.00	14.00	33.33%***	49.78	7.78	18.52%
Oral	24.00	14.00	-10.00	-41.67%	32.28	8.28	34.51%***	27.38	3.38	14.07%***
Orientation	24.00	50.17	26.17	109.03%***	52.05	28.05	116.86%***	49.78	25.78	107.41%***
Mobility	30.00	45.50	15.50	51.67%**	52.71	22.71	75.69%***	49.78	19.78	65.93%***
Social	20.00	42.00	22.00	110.00%***	54.02	34.02	170.12%***	39.82	19.82	99.11%***
Nausea	25.00	35.00	10.00	40.00%	51.39	26.39	105.55%***	49.78	24.78	99.11%***
Appetite	37.00	39.67	2.67	7.21%	53.36	16.36	44.23%***	53.51	16.51	44.62%**
Medication	49.00	47.83	-1.17	-2.38%	54.68	5.68	11.60%***	49.78	0.78	1.59%
Elimination	35.00	45.50	10.50	30.00%	53.36	18.36	52.47%**	52.27	17.27	49.33%**
**Overall Mean**	**31.78**	**40.83**	**9.06**	28.50%*	**51.10**	**19.32**	60.79%***	**46.87**	**15.10**	47.51%**

***p < .001, **p < .01, *p < .05

At one month (t = 1), the assessment and documentation of all domains, except two – namely, oral and medication – improved. Particular improvements were demonstrated in the assessment and documentation of a patient’s social situation (110.00%, *p* < .001) and orientation (109.03%, *p* < .001) – these are noteworthy given that: social needs often remain unmet among patients who require palliative care and their carers [39]; and assessing orientation can be challenging [40]. It is curious that the assessment and documentation of both oral and medication changes declined during this timeframe (-41.67% and − 2.4%, respectively). While determining explanation(s) was beyond the scope of this project, the low occurrences for the oral health domain might suggest the availability of few protocols to guide oral care for patients who require palliative care [41]. Additionally, the medication domain might be partly because the baseline figure for this domain was initially relatively high, documented in 87.5% of cases – as such, there was less room for improvement. Nevertheless, the aide-mémoire appears to have improved assessment and documentation overall in the short-term.

At eight months (t = 8), the initial improvements were sustained. Relative to the baseline measures and the measures at one month (t = 1), the assessment and documentation of all domains significantly improved (*p* < .01) – this was particularly the case regarding, once again, patients’ orientation (116.86%) and social situation (170.12%), but also included nausea (105.55%). All but one of the domains (oral health) were documented in over 90% of the case notes. The results indicate the aide-mémoire substantially improved assessment and documentation in the medium-term.

The improvements were largely sustained in the long-term. At fifty months (t = 50), the assessment and documentation of all domains had improved, relative to the baseline measure. This was particularly the case for orientation (107.41%), social (99.11%), and nausea (99.11%).

**Fig. 2:**
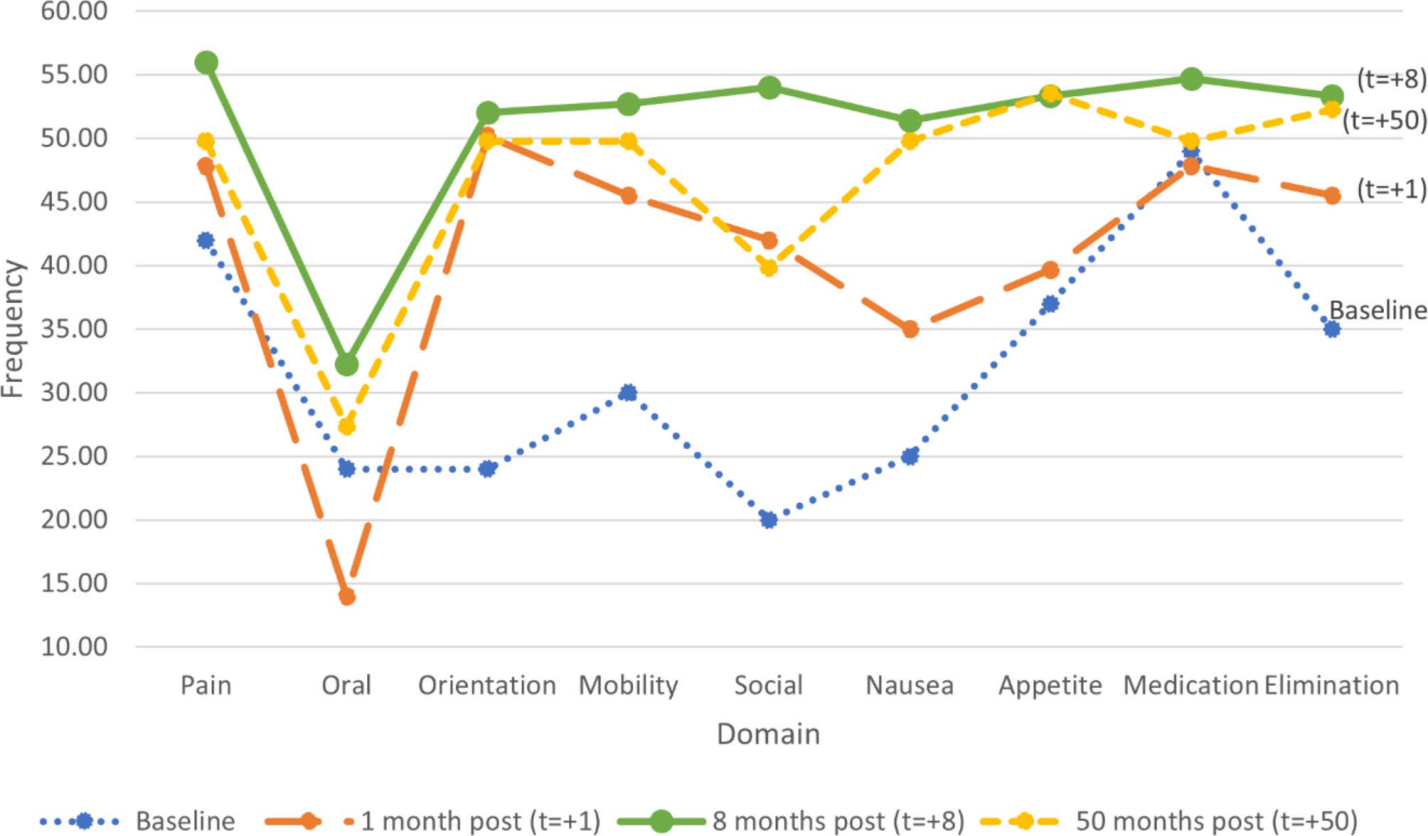
Occurrence of domains documented in case notes over time

In the short-, medium-, and long-term, the assessment and documentation of almost all of the palliative care domains improved, following the introduction of the aide-mémoire (see Fig. 2). The greatest improvement across all domains was at eight months (t = 8), with improvement rates slightly declining in the period between eight and fifty months. These findings suggest the value of reminding and supporting clinicians to continue using the aide-mémoire; they also reflect research on the benefits of reflective practice, lifelong learning, and service evaluation for continued improvement in the quality of palliative care [22, 42, 43].

## Discussion

Assessment and documentation skills are pivotal to quality palliative care – this extends to community-based palliative care [44]. As demonstrated in this article, the aide-mémoire – POMSNAME – represents an effective way to improve clinician assessment and documentation of important palliative care domains. These include a patient’s: pain, orientation and oral health, mobility, social situation, nausea, appetite, medication, and elimination. Specifically, this longitudinal evaluation of community health nurses’ patient case notes found improved assessment and documentation of all domains in the medium-term and in the long-term, with the most significant improvement across all domains in the medium term (at *p* < .001 and *p* < .01 levels).

The aforesaid findings are noteworthy for two key reasons. First, it demonstrates the sustained impact of an inexpensive resource – an aide-mémoire – which was informed by extant literature [3, 8, 26, 27, 33, 34, 45] and supported by formal and informal training. Reflecting literature on innovation [46–48], this might be partly due to: its user-friendliness – as it was presented to the community health nurses as a guide to be adapted for use, rather than a directive; its accessibility, whereby the community health nurses had ready access to the placard; and the ongoing support for its implementation. Second, given that palliative care is everyone’s business [49, 50], the findings demonstrate a viable way to promote quality palliative care among those with generalist expertise, thereby addressing an establishing need [51]. Given the demonstrated value of the aide-mémoire, it was subsequently endorsed by the Primary and Community Health Quality and Safety Committee for use throughout the primary and community health services within the local health district. Furthermore, team members were awarded a Quality Award to recognise this innovation within the local health district.

Despite the value of the findings, four methodological limitations warrant mention. First, given the project involved a small number of community health nurses affiliated with one community health centre, there are no claims that the findings can be generalised to the contexts, within or beyond Australia or professions. Second, as an evaluation of patient case notes, it was not feasible to verify clinician practices during patient consultations – as such, the case notes were assumed to reflect clinician practices. The analysis of additional data sourced via observational methods might assist in verifying these practices. Third, although all case notes within the project period were evaluated, and despite the involvement of auditors who did not provide palliative care to patients whose case notes were evaluated, the results might be biased, given that: the case notes were not randomly evaluated; and nurses who provided palliative care to the patients collated and contributed to the evaluation. Fourth, because the case notes were not documented at the same timepoints as patient illness progressed, particular concerns might have been more (or less) salient at the time the clinicians documented the case notes – this might have influenced the matters that clinicians documented.

## Conclusion

This project demonstrates promising results that warrant further consideration. This would involve addressing the aforesaid limitations in the first instance and, pending the associated findings, testing the aide-mémoire in other palliative care services. The findings have important implications for scholars and clinicians, alike. For scholars, research is needed to clarify: why the greatest improvement was at eight months (t = 8); why this level of improvement was not sustained; what helps and hinders clinician use of resources that aim to improve assessment and documentation, in situ; the forms and regularity of formal and informal training that are likely to be (un)helpful; patient and carer perceptions of, and experiences with these resources; and how adversity and crises – such as COVID-19 – influence resource use. For clinicians, there is considerable opportunity to: trial the aide-mémoire in different contexts with different professions; and consider which domains also warrant inclusion (e.g., sexual health, skin integrity, spirituality, etc.) and how. Given the important roles of assessment and documentation in quality palliative care [44], this modest project represents a worthwhile contribution to the evidence on how to improve palliative care, particularly that which is community-based.

### Electronic supplementary material

Below is the link to the electronic supplementary material.

Supplementary Material 1

## Data Availability

The dataset analysed during the current study is not publicly available given the nature of the project but are available from the corresponding author on reasonable request.

## References

[1] Vernon E, Hughes MC, Kowalczyk M (2022). Measuring effectiveness in community-based palliative care programs: a systematic review. Soc Sci Med.

[2] Spencer S, Gomez S, Silbermann M (2021). Models of community-based palliative care. Palliative care for chronic cancer patients in the community.

[3] Mistry B, Bainbridge D, Bryant D, Tan Toyofuku S, Seow H (2015). What matters most for end-of-life care? Perspectives from community-based palliative care providers and administrators. BMJ Open.

[4] Chirico J, Donnelly JP, Gupton A, Cromwell P, Miller M, Dawson C (2019). Costs of care and location of death in community-based pediatric palliative care. J Palliat Med.

[5] Murakami N, Tanabe K, Morita T, Fujikawa Y, Koseki S, Kajiura S (2018). Impact of a six-year project to enhance the awareness of community-based palliative care on the place of death. J Palliat Med.

[6] Fee A, Muldrew D, Slater P, Payne S, McIlfatrick S, McConnell T (2020). The roles, responsibilities and practices of healthcare assistants in out-of-hours community palliative care: a systematic scoping review. Palliat Med.

[7] Yosick L, Crook REm, Gatto M, Maxwell TL, Duncan I, Ahmed T (2019). Effects of a population health community-based palliative care program on cost and utilization. J Palliat Med.

[8] Goni-Fuste B, Crespo I, Monforte-Royo C, Porta-Sales J, Balaguer A, Pergolizzi D (2021). What defines the comprehensive assessment of needs in palliative care? An integrative systematic review. Palliat Med.

[9] Sjöberg M, Edberg AK, Rasmussen BH, Beck I (2021). Documentation of older people’s end-of-life care in the context of specialised palliative care: a retrospective review of patient records. BMC Palliat Care.

[10] Natuhwera G, Rabwoni M, Ellis P, Merriman A (2021). Clinicians’ and nurses’ documentation practices in palliative and hospice care. Int J Palliat Nurs.

[11] Bruera E, Kuehn N, Miller MJ, Selmser P, Macmillan K (1991). The Edmonton symptom assessment system (ESAS): a simple method for the assessment of palliative care patients. J Palliat Care.

[12] Saleem TZ, Higginson IJ, Chaudhuri KR, Martin A, Burman R, Leigh PN (2012). Symptom prevalence, severity and palliative care needs assessment using the Palliative Outcome Scale: a cross-sectional study of patients with Parkinson’s disease and related neurological conditions. Palliat Med.

[13] Yun YH, Kang EK, Lee J, Choo J, Ryu H, Yun HM (2018). Development and validation of the quality care questionnaire - palliative care (QCQ-PC): patient-reported assessment of quality of palliative care. BMC Palliat Care.

[14] Masel EK, Berghoff AS, Schur S, Maehr B, Schrank B, Simanek R (2016). The PERS2ON score for systemic assessment of symptomatology in palliative care: a pilot study. Eur J Cancer Care.

[15] de Lima AC, Arantes Q (2021). Clinical assessment of human suffering: planning care in the end of life.

[16] Wagner A, Schaffert R, Möckli N, Zúñiga F, Dratva J (2020). Home care quality indicators based on the resident assessment instrument-home care (RAI-HC): a systematic review. BMC Health Serv Res.

[17] Seccareccia D, Wentlandt K, Kevork N, Workentin K, Blacker S, Gagliese L (2015). Communication and quality of care on palliative care units: a qualitative study. J Palliat Med.

[18] Kremeike K, Dojan T, Rosendahl C, Jünger S, Romotzky V, Boström K (2021). Withstanding ambivalence is of particular importance - controversies among experts on dealing with desire to die in palliative care. PLoS ONE.

[19] Pastrana T, Wüller J, Weyers S, Bruera E (2021). Insights from a community-based palliative care course: a qualitative study. BMC Palliat Care.

[20] Jiao K, Chow AY, Wang J, Chan II (2021). Factors facilitating positive outcomes in community-based end-of-life care: a cross-sectional qualitative study of patients and family caregivers. Palliat Med.

[21] Ding J, Johnson CE, Saunders C, Licqurish S, Chua D, Mitchell G (2022). Provision of end-of-life care in primary care: a survey of issues and outcomes in the australian context. BMJ Open.

[22] Slater PJ, Osborne CJ, Herbert AR (2021). Ongoing value and practice improvement outcomes from pediatric palliative care education: the quality of care collaborative Australia. Adv Med Educ Pract.

[23] Ogrinc G, Davies L, Goodman D, Batalden P, Davidoff F, Stevens D (2016). SQUIRE 2.0 (Standards for quality improvement reporting excellence): revised publication guidelines from a detailed consensus process. BMJ Qual Saf.

[24] Wattanapaiboon K, Banditlerdruk S, Vattanavanit V (2020). Presenting symptoms in sepsis: is the mnemonic SEPSIS useful?. Infect Drug Resist.

[25] Tulaimat A, Trick WE, DiapHRaGM (2017). A mnemonic to describe the work of breathing in patients with respiratory failure. PLoS ONE.

[26] Daveson BA, Allingham SF, Clapham S, Johnson CE, Currow DC, Yates P (2021). The PCOC symptom assessment scale (SAS): a valid measure for daily use at point of care and in palliative care programs. PLoS ONE.

[27] Iaboni A, Van Ooteghem K, Marcil MN, Cockburn A, Flint AJ, Grossman D (2018). A palliative approach to falls in advanced dementia. Am J Geriatr Psychiatry.

[28] Wilberg P, Hjermstad MJ, Ottesen S, Herlofson BB (2012). Oral health is an important issue in end-of-life cancer care. Support Care Cancer.

[29] Venkatasalu MR, Murang ZR, Ramasamy DTR, Dhaliwal JS (2020). Oral health problems among palliative and terminally ill patients: an integrated systematic review. BMC Oral Health.

[30] Richardson A, Medina J, Brown V, Sitzia J (2007). Patients’ needs assessment in cancer care: a review of assessment tools. Support Care Cancer.

[31] Ellershaw JE, Peat SJ, Boys LC (1995). Assessing the effectiveness of a hospital palliative care team. Palliat Med.

[32] Cadogan CA, Murphy M, Boland M, Bennett K, McLean S, Hughes C (2021). Prescribing practices, patterns, and potential harms in patients receiving palliative care: a systematic scoping review. Exploratory Res Clin Social Pharm.

[33] Muldrew DHL, Hasson F, Carduff E, Clarke M, Coast J, Finucane A (2018). Assessment and management of constipation for patients receiving palliative care in specialist palliative care settings: a systematic review of the literature. Palliat Med.

[34] Hall A, Ewing G, Rowland C, Grande G (2020). A drive for structure: a longitudinal qualitative study of the implementation of the carer support needs assessment tool (CSNAT) intervention during hospital discharge at end of life. Palliat Med.

[35] ABS (Australian Bureau of Statistics). Sydney - South West [Website]. Belconnen, ACT: ABS (Australian Bureau of Statistics); n.d. [Available from: https://www.abs.gov.au/census/find-census-data/quickstats/2016/127.

[36] NSW Health. South Western Sydney [Website]. St Leonards, NSW: NSW Health; n.d. [Available from: https://www.health.nsw.gov.au/lhd/Pages/swslhd.aspx#:~:text=SWSLHD%20covers%20seven%20Local%20Government,palliative%20care%20and%20rehabilitation%20services.

[37] NHMRC (National Health and Medical Research Council) (2014). Ethical considerations in quality assurance and evaluation activities.

[38] WHO (World Health Organization) (2021). Assessing the development of palliative care worldwide: a set of actionable indicators.

[39] Ventura AD, Burney S, Brooker J, Fletcher J, Ricciardelli L (2014). Home-based palliative care: a systematic literature review of the self-reported unmet needs of patients and carers. Palliat Med.

[40] Gonçalves F, Bento MJ, Alvarenga M, Costa I, Costa L (2008). Validation of a consciousness level scale for palliative care. Palliat Med.

[41] Kong A, George A, Villarosa A, Srinivas R, Agar M, Harlum J (2020). Perceptions of nurses towards oral health in palliative care: a qualitative study. Collegian.

[42] Rosenberg ME (2018). An outcomes-based approach across the medical education continuum. Trans Am Clin Climatol Assoc.

[43] Jones R (2019). Medical education research: evidence, evaluation and experience. Educ Prim Care.

[44] Williams N, Boumans N, Luymes N, White NE, Lemonde M, Guthrie DM (2022). What should be measured to assess the quality of community-based palliative care? Results from a collaborative expert workshop. Palliat Support Care.

[45] Ahmed N, Ahmedzai SH, Collins K, Noble B (2014). Holistic assessment of supportive and palliative care needs: the evidence for routine systematic questioning. BMJ Supportive & Palliative Care.

[46] Rogers EM (2003). Diffusion of innovations.

[47] Greenhalgh T, Robert G, Macfarlane F, Bate P, Kyriakidou O (2004). Diffusion of innovations in service organizations: systematic review and recommendations. Milbank Q.

[48] Parmar J, Sacrey LA, Anderson S, Charles L, Dobbs B, McGhan G (2022). Facilitators, barriers and considerations for the implementation of healthcare innovation: a qualitative rapid systematic review. Health Soc Care Community.

[49] Pereira J, Chasen MR (2016). Early palliative care: taking ownership and creating the conditions. Curr Oncol.

[50] Sansom-Daly UM, Lobb EA, Evans HE, Breen LJ, Ugalde A, Best M (2021). To be mortal is human: Professional consensus around the need for more psychology in palliative care. BMJ Supportive & Palliative Care.

[51] Alshammari F, Sim J, Lapkin S, Stephens M (2022). Registered nurses’ knowledge, attitudes and beliefs about end-of-life care in non-specialist palliative care settings: a mixed studies review. Nurse Educ Pract.

