# Supplementary material for: POMSNAME: an aide-mémoire to improve the assessment and documentation of palliative care – a longitudinal project
Source: BMC Palliat Care. 2023 Oct 21;22:157. doi: 10.1186/s12904-023-01279-1 (PMC10590006; doi:10.1186/s12904-023-01279-1)
Supplement: Supplementary file 1 — Supplementary Material 1 [file 12904_2023_1279_MOESM1_ESM.docx]

Revised Standards for Quality Improvement Reporting Excellence

SQUIRE 2.0

| Title and Abstract |  | Authors’ Response |
| --- | --- | --- |
| 1. Title | Indicate that the manuscript concerns an [initiative](http://www.squire-statement.org/index.cfm?fuseaction=page.viewpage&pageid=485#Initiative) to improve healthcare (broadly defined to include the quality, safety, effectiveness, patient-centeredness, timeliness, cost, efficiency, and equity of healthcare) | This manuscript focuses on a memory aide to improve the assessment and documentation of palliative care. |
| 1. Abstract | 1. Provide adequate information to aid in searching and indexing 2. Summarize all key information from various sections of the text using the abstract format of the intended publication or a structured summary such as: background, local problem, methods, interventions, results, conclusions | The abstract was developed to optimise searching and indexing efforts and summarise all key information from various sections of the text in a structured manner. |
| Introduction | Why did you start? |  |
| 1. [Problem Description](http://www.squire-statement.org/index.cfm?fuseaction=page.viewpage&pageid=485#Problem) | Nature and significance of the local [problem](http://www.squire-statement.org/index.cfm?fuseaction=page.viewpage&pageid=485#Problem) | The nature and significance of the local problem was explicated. |
| 1. Available Knowledge | Summary of what is currently known about the [problem](http://www.squire-statement.org/index.cfm?fuseaction=page.viewpage&pageid=485#Problem), including relevant previous studies | International evidence was reviewed to contextualise the problem within the evidence-base. |
| 1. Rationale | Informal or formal frameworks, models, concepts, and/or [theories](http://www.squire-statement.org/index.cfm?fuseaction=page.viewpage&pageid=485#Theory) used to explain the [problem](http://www.squire-statement.org/index.cfm?fuseaction=page.viewpage&pageid=485#Problem), any reasons or [assumptions](http://squire.citysoft.org/index.cfm?fuseaction=page.viewPage&pageID=485&nodeID=1#assumptions) that were used to develop the [intervention(s),](http://www.squire-statement.org/index.cfm?fuseaction=page.viewpage&pageid=485#Interventions)and reasons why the [intervention(s)](http://www.squire-statement.org/index.cfm?fuseaction=page.viewpage&pageid=485#Interventions) was expected to work | A rationale for the memory aide was duly provided, with reference to relevant academic literature. |
| 1. Specific Aims | Purpose of the project and of this report | The aim of the project was clearly indicated. |
| Methods | What did you do? |  |
| 1. [Context](http://www.squire-statement.org/index.cfm?fuseaction=page.viewpage&pageid=485#context) | Contextual elements considered important at the outset of introducing the [intervention(s)](http://www.squire-statement.org/index.cfm?fuseaction=page.viewpage&pageid=485#Interventions) | The context was described. |
| 1. [Intervention(s)](http://www.squire-statement.org/index.cfm?fuseaction=page.viewpage&pageid=485#Interventions) | 1. Description of the [intervention(s)](http://www.squire-statement.org/index.cfm?fuseaction=page.viewpage&pageid=485#Interventions) in sufficient detail that others could reproduce it 2. Specifics of the team involved in the work | The memory aide was described, justified, and illustrated – furthermore, the team involved was described. |
| 1. Study of the Intervention(s) | 1. Approach chosen for assessing the impact of the [intervention(s)](http://www.squire-statement.org/index.cfm?fuseaction=page.viewpage&pageid=485#Interventions) 2. Approach used to establish whether the observed outcomes were due to the [intervention(s)](http://www.squire-statement.org/index.cfm?fuseaction=page.viewpage&pageid=485#Interventions) | The evaluation methods were described to gauge the effects associated with the memory aide. |
| 1. Measures | 1. Measures chosen for studying [processes](http://www.squire-statement.org/index.cfm?fuseaction=page.viewpage&pageid=485#Process) and outcomes of the [intervention(s),](http://www.squire-statement.org/index.cfm?fuseaction=page.viewpage&pageid=485#Interventions)including rationale for choosing them, their operational definitions, and their validity and reliability 2. Description of the approach to the ongoing assessment of contextual elements that contributed to the success, failure, efficiency, and cost 3. Methods employed for assessing completeness and accuracy of data | The measures were described and justified. |
| 1. Analysis | 1. Qualitative and quantitative methods used to draw [inferences](http://www.squire-statement.org/index.cfm?fuseaction=page.viewpage&pageid=485#Inferences) from the data 2. Methods for understanding variation within the data, including the effects of time as a variable | The analysis of quantitative data was described and justified. |
| 1. Ethical Considerations | [Ethical aspects](http://www.squire-statement.org/index.cfm?fuseaction=page.viewpage&pageid=485#Ethical_aspects)of implementing and studying the [intervention(s)](http://www.squire-statement.org/index.cfm?fuseaction=page.viewpage&pageid=485#Interventions) and how they were addressed, including, but not limited to, formal ethics review and potential conflict(s) of interest | As a quality improvement project that met the national definition of quality assurance and evaluation^1^, the approval of a human research ethics committee was not required. |
| Results | What did you find? |  |
| 1. Results | 1. Initial steps of the [intervention(s)](http://www.squire-statement.org/index.cfm?fuseaction=page.viewpage&pageid=485#Interventions) and their evolution over time (e.g., time-line diagram, flow chart, or table), including modifications made to the intervention during the project 2. Details of the [process](http://www.squire-statement.org/index.cfm?fuseaction=page.viewpage&pageid=485#Process) measures and outcome 3. Contextual elements that interacted with the [intervention(s)](http://www.squire-statement.org/index.cfm?fuseaction=page.viewpage&pageid=485#Interventions) 4. Observed associations between outcomes, interventions, and relevant contextual elements 5. Unintended consequences such as unexpected benefits, [problems](http://www.squire-statement.org/index.cfm?fuseaction=page.viewpage&pageid=485#Problem), failures, or costs associated with the [intervention(s)](http://www.squire-statement.org/index.cfm?fuseaction=page.viewpage&pageid=485#Interventions) 6. Details about missing data | The steps were tabulated for ease of reference and the absence of missing data was noted. Furthermore, the results associated with the memory aide were explicated. |
| Discussion | What does it mean? |  |
| 1. Summary | 1. Key findings, including relevance to the [rationale](http://www.squire-statement.org/index.cfm?fuseaction=page.viewpage&pageid=485#Rationale) and specific aims 2. Particular strengths of the project | The key findings were summarised and their importance were noted. |
| 1. Interpretation | 1. Nature of the association between the [intervention(s)](http://www.squire-statement.org/index.cfm?fuseaction=page.viewpage&pageid=485#Interventions) and the outcomes 2. Comparison of results with findings from other publications 3. Impact of the project on people and [systems](http://www.squire-statement.org/index.cfm?fuseaction=page.viewpage&pageid=485#Systems) 4. Reasons for any differences between observed and anticipated outcomes, including the influence of [context](http://www.squire-statement.org/index.cfm?fuseaction=page.viewpage&pageid=485#context) 5. Costs and strategic trade-offs, including [opportunity costs](http://www.squire-statement.org/index.cfm?fuseaction=page.viewpage&pageid=485#Opportunity_costs) | The key findings were interpreted with reference to relevant literature. |
| 1. Limitations | 1. Limits to the [generalizability](http://www.squire-statement.org/index.cfm?fuseaction=page.viewpage&pageid=485#Generalizability) of the work 2. Factors that might have limited [internal validity](http://www.squire-statement.org/index.cfm?fuseaction=page.viewpage&pageid=485#Internal_validity) such as confounding, bias, or imprecision in the design, methods, measurement, or analysis 3. Efforts made to minimize and adjust for limitations | The limitations were explicated. |
| 1. Conclusions | 1. Usefulness of the work 2. Sustainability 3. Potential for spread to other [contexts](http://www.squire-statement.org/index.cfm?fuseaction=page.viewpage&pageid=485#context) 4. Implications for practice and for further study in the field 5. Suggested next steps | The usefulness of the work and the implications for clinicians and scholars were duly explicated. |
| Other Information |  |  |
| 1. Funding | Sources of funding that supported this work. Role, if any, of the funding organization in the design, implementation, interpretation, and reporting | The funding source was duly acknowledged. |

# References

1. NHMRC (National Health and Medical Research Council). *Ethical considerations in quality assurance and evaluation activities*. 2014. Canberra, ACT: NHMRC (National Health and Medical Research Council).
